# Supplementary material for: Causal Relationship between Aging and Anorexia Nervosa: A White-Matter-Microstructure-Mediated Mendelian Randomization Analysis
Source: Biomedicines. 2024 Aug 16;12(8):1874. doi: 10.3390/biomedicines12081874 (PMC11351342; doi:10.3390/biomedicines12081874)
Supplement: Supplementary file 1 [file biomedicines-12-01874-s001.zip › Supplementary Figure/Figure S4/Singe SNP plot of MR analysis for TL to ubm-b-1846.pdf]

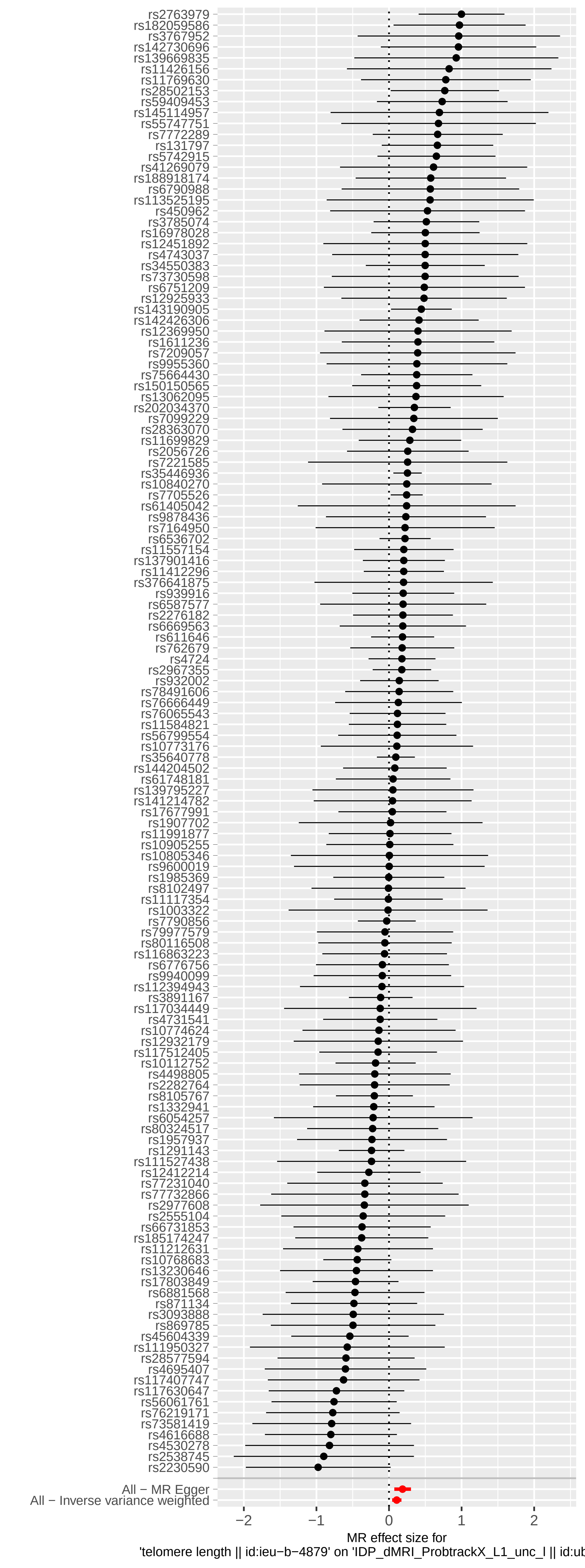

rs2763979
rs182059586
rs3767952
rs142730696
rs139669835
rs11426156
rs11769630
rs28502153
rs59409453
rs145114957
rs55747751
rs7772289
rs131797
rs5742915
rs41269079
rs188918174
rs6790988
rs113525195
rs450962
rs3785074
rs16978028
rs12451892
rs4743037
rs34550383
rs73730598
rs6751209
rs12925933
rs143190905
rs142426306
rs12369950
rs1611236
rs7209057
rs9955360
rs75664430
rs150150565
rs13062095
rs202034370
rs7099229
rs28363070
rs11699829
rs2056726
rs7221585
rs35446936
rs10840270
rs7705526
rs61405042
rs9878436
rs7164950
rs6536702
rs11557154
rs137901416
rs11412296
rs376641875
rs939916
rs6587577
rs2276182
rs6669563
rs611646
rs762679
rs4724
rs2967355
rs932002
rs78491606
rs76666449
rs76065543
rs11584821
rs56799554
rs10773176
rs35640778
rs144204502
rs61748181
rs139795227
rs141214782
rs17677991
rs1907702
rs11991877
rs10905255
rs10805346
rs9600019
rs1985369
rs8102497
rs11117354
rs1003322
rs7790856
rs79977579
rs80116508
rs116863223
rs6776756
rs9940099
rs112394943
rs3891167
rs117034449
rs4731541
rs10774624
rs12932179
rs117512405
rs10112752
rs4498805
rs2282764
rs8105767
rs1332941
rs6054257
rs80324517
rs1957937
rs1291143
rs111527438
rs12412214
rs77231040
rs77732866
rs2977608
rs2555104
rs66731853
rs185174247
rs11212631
rs10768683
rs13230646
rs17803849
rs6881568
rs871134
rs3093888
rs869785
rs45604339
rs111950327
rs28577594
rs4695407
rs117407747
rs117630647
rs56061761
rs76219171
rs73581419
rs4616688
rs4530278
rs2538745
rs2230590
All − MR Egger
All − Inverse variance weighted
−2
−1
0
1
2
MR effect size for
'telomere length || id:ieu−b−4879' on 'IDP_dMRI_ProbtrackX_L1_unc_l || id:ub
